# Why are some outbreaks worse than others? COVID-19 outbreak management strategies from a PHU perspective

**DOI:** 10.1186/s12889-023-15498-x

**Published:** 2023-03-30

**Authors:** Emma Hodge, Shannen Oversby, Josette Chor

**Affiliations:** Wide Bay Public Health Unit (WBPHU), Queensland Health, Hervey Bay, Australia

**Keywords:** COVID-19, Outbreaks, Aged care facilities, Public health units

## Abstract

**Background:**

From a Public Health Unit (PHU) perspective, this review aimed to examine factors associated with adverse outbreak outcomes, to identify evidence based focal strategies of managing COVID-19 outbreaks in aged care settings.

**Methods:**

A retrospective review of PHU documentation examined all 55 COVID-19 outbreaks in Wide Bay RACFs across the first 3 COVID-19 waves in Queensland, through thematic and statistical analysis. ​.

**Results:**

Thematic analysis using the framework approach identified 5 themes associated with outcomes of COVID-19 outbreaks in RACFs. These were analysed for statistical significance against outbreak outcomes including duration, attack rate and case fatality rate. There was a significant relationship between memory support unit (MSU) involvement and adverse outbreak outcomes. Attack rate was significantly associated with communication frequency, symptom monitoring and case detection approach, staff shortages and cohorting. Staff shortages were also significantly associated with a prolonged outbreak duration. There was no statistically significant relationship between outbreak outcomes and resource availability or infection control strategy. ​.

**Conclusions:**

This emphasises the importance of frequent communication between PHUs and RACFs during active outbreaks, as well as the need for regular symptom monitoring and prompt case detection, to minimise viral transmission. Staff shortages and cohorting are also crucial factors to be addressed during outbreak management.

**Implications for Public Health:**

This review adds to the evidence basis of COVID-19 outbreak management strategies to improve PHU advice to RACFs, to mitigate viral transmission and ultimately reduce the burden of disease associated with COVID-19 and other communicable diseases.

## Background

From 10 million positive COVID-19 cases as of September 2022, [1, 2] Australia reported a cumulative total of 8132 outbreaks across 2724 residential aged care facilities (RACFs), including 6066 and 92,424 cases in staff and residents respectively. [3]

RACFs are high risk settings for COVID-19 due to both the health vulnerabilities associated with age and underlying medical comorbidities, as well as the density of living arrangements. [4] Despite the critical intervention of vaccination, COVID-19 remains a severe disease for some, especially the elderly [5] and aged care residents face a greater mortality rate of just over 4% compared to 0.1% in the general population [5–7]. Deaths in residential aged care account for around 30% of all reported COVID-19 deaths in Australia, which is among the highest rates worldwide. [5, 8–10]

COVID-19 outbreaks pose a significant burden aged care facility. Collateral consequences for residents include reduced visitation, group activities, physical decline and implications on mental health [11–13], while aged care workers are dealt an increased workload in the context of prepandemic staff shortages across the aged care sector [14, 15]. COVID-19 and influenza-like illness outbreaks also impact the healthcare system, as large numbers of elderly infections beget an increase in hospitalisations in the context of an already strained health service. [16]

With over 200,000 people in residential aged care facilities, resourcing for this sector affects a significant number of Australians. [8] Aged care facilities require significant support from multiple stakeholders to manage COVID-19 outbreaks including Public Health Units (PHU), Commonwealth Department of Health, Aged Care Quality and Safety Commission, Residential Aged Care Facility Support Service (RaSS) and medical follow up via General Practitioners or the Hospital and Health Service COVID-19 virtual ward.

Prior to the COVID-19 pandemic, PHUs have a long history of involvement in communicable disease control within RACFs, such as seasonal influenza and gastroenteritis outbreak management. PHUs have therefore played a central role during the COVID-19 pandemic in Australia, coordinating the response to high-risk outbreaks and providing advice to multiple stakeholders. While there are state and national guidelines on RACF COVID-19 outbreak management, each facility has their own policy and protocols.

Outbreak management guidelines have continued to evolve, especially during 2022, due to changing evidence, risk tolerance and the SARS-CoV-2 virus itself. During the early periods of COVID-19 transmission, RACFs faced strict lockdowns and isolation of close contacts. Now more than two years into the pandemic, the Chief Health Officer (CHO) direction permits free movement of close contacts and RACFs seldom isolate residents to their room unless a confirmed case. [17, 18] Gradual easing of public health restrictions in Australia have proliferated the number of RACF outbreaks, with three main peak periods of COVID-19 transmission in Queensland to date, commonly referred to as waves. [19]

There is a dearth of literature to explain why some COVID-19 outbreaks are associated with greater adverse outcomes than others. There is profound variability in outbreak duration, attack rate and case fatality rate across aged care settings. [5, 9–11] With future SARS-COV-2 variants expected to arise and associated ongoing outbreaks, the need to effectively manage COVID-19 and respiratory illnesses in aged care facilities remains paramount to prevent morbidity and mortality. [20] It is therefore crucial to evaluate the outbreaks across the three COVID-19 waves to date and the barriers and enablers to their management.

From a PHU perspective, we aim to identify factors associated with adverse outbreak outcomes to provide evidence-based strategies in outbreak management advice. By reviewing evidence underpinning the variability in outbreak outcomes, our findings intend to guide evidence-based management of COVID-19 and other respiratory virus outbreaks, to ultimately reduce the burden of viral transmission with RACFs.

## Methods

### Study Design and setting

Wide Bay Public Health Unit (WBPHU) is situated in Wide Bay, a region of Queensland located between 170 and 400 km north of Brisbane with a population of over 300,000, comprising the regional cities of Bundaberg, Hervey Bay and Maryborough, as well as small rural towns. [21]

The study design was a retrospective audit of existing PHU documentation, comprising data pertaining to COVID-19 outbreaks in Wide Bay RACFs.

### Population and participants

The source population included all 28 RACFs within the catchment of the WBPHU, excluding multi-purpose health services with 36 aged care beds managed by Wide Bay Hospital and Health Service. [21] Data was collected on 27 RACFs who met the following definition of a COVID-19 outbreak between 01 and 2022 and 31 August 2022, based on most recent definition published by the Communicable Disease Network Australia (CDNA) on the 15th of February 2022.

- Two or more residents of a RACF who have been diagnosed with COVID-19 via RAT (rapid antigen test) or PCR (polymerase chain reaction) test within 5 days and have been onsite at the RACF at any time during their infectious period: or.
- Five or more staff, visitors and/or residents of the RACF diagnosed with COVID-19 through RAT or PCR test within the past 7 days who worked/visited during their infectious period.

RACFs notify COVID-19 outbreaks to the PHU and Commonwealth. Cases were identified from existing PHU records and therefore only includes outbreaks which were reported.

### Outbreak definition

The outbreak start date was defined as the day when the CDNA outbreak criteria in the RACF was met, rather than the day of the first COVID-19 positive case. The end date is defined in CDNA guideline as 7 days since the last case in the facility. At the time of analysis, the isolation period for confirmed cases of COVID-19 was 7 days. [17] All outbreaks were divided into three time periods based on the three COVID-19 waves as defined by the following dates:

- 1st wave: 1 January 2022 to 13 March 2022 [BA.1].
- 2nd wave: 14 March to 12 June 2022 [BA.2].
- 3rd wave: 13 June – 31 August 2022 [BA.5].

### Data collection

Qualitative and quantitative data was collected retrospectively from clinical documentation written by WBPHU staff during RACF outbreaks, including clinical notes, facility outbreak line lists, PHU phone records, emails and meeting minutes. This included records of all correspondence between the PHU and RACF including resource availability, approach to case detection, staff cohorting, staff shortages and infection control strategies. Specific outbreak details such as start and end date, number of staff and resident cases.

The WBPHU used spreadsheets to capture all outbreak data which was saved to a password protected Microsoft Teams file and all researchers had access to this through their role at the PHU at the time of data collection.

Total resident and staff numbers were available from existing PHU records and was confirmed with details on facility outbreak line lists to verify denominators. Resident deaths from COVID-19 were obtained from facility line lists.

All WBPHU records were reviewed by the principal investigator in relation to RACF COVID-19 outbreaks during the specified time period. RACFs were assigned individual identification numbers unique to this project and de-identified data was entered directly into a password protected spreadsheet.

### Quantitative analysis

STATA version 17 was used for all statistical analysis and to generate summary statistics.

Outbreak outcomes were defined as outbreak duration, attack rate (residents, staff and total), CFR and number of outbreaks. These variables were assessed for normality with a visual inspection of a histogram and through skewness and kurtosis tests. As skewness was significant for all variables, median and interquartile range were used to summarise results and analysis was conducted using non-parametric tests. Spearman’s correlation was used to evaluate the relationship between facility size and outbreak outcomes.

Outbreak outcomes were analysed across each of the three COVID-19 waves, as well as the effect of binary variables; index case, baseline personal protective equipment (PPE) and memory support unit (MSU) involvement using Kruskal-Wallis tests. [22] The threshold for statistical significance was set at p < 0.05.

### Qualitative analysis

Thematic analysis was performed on qualitative data using the Framework method [23]. The principal investigator (Author EH) reviewed the documentation and generated initial codes. Two investigators (Authors EH and SO) then reviewed the codes independently to mitigate conscious and unconscious bias in data selection and identified common codes which were grouped together into themes. There were very few discrepancies, and these were resolved through consensus by refining theme names and merging or omitting duplications. Themes were reviewed again with the original data and grouped together to incorporate subthemes. Themes were not classified as barriers or enablers during the data collection stage to further minimise bias in selection.

**Fig. 1:**
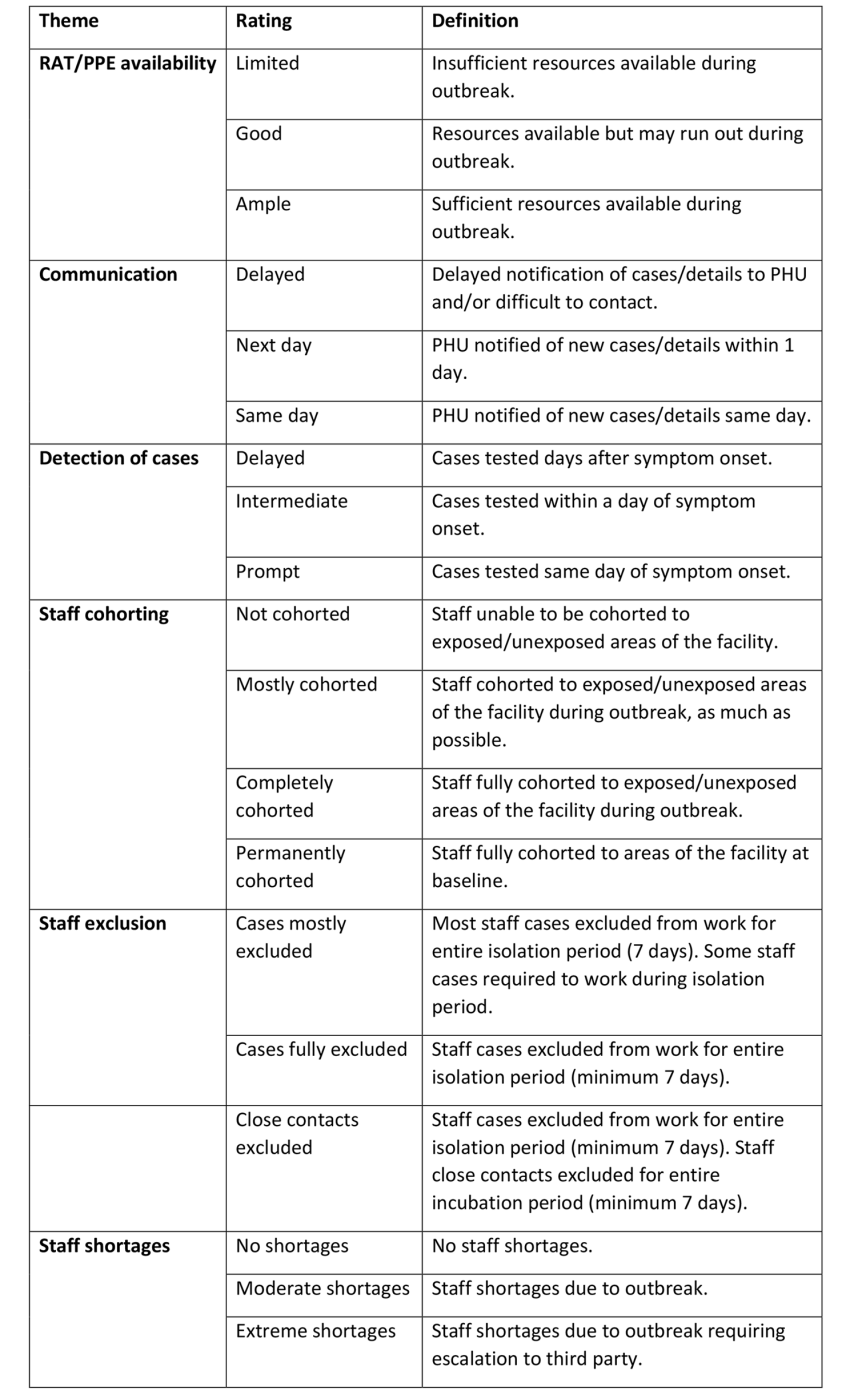
Theme definitions

Once the analytical framework was established, 4 themes were selected for further analysis due to aspects of measurable, objective classification. 2 themes were omitted from this component of analysis due to the risk of subjectivity in interpreting documentation to provide a rating. The authors developed an objective rating system for theme and re-reviewed the documentation to score each outbreak. Figure 1 outlines the definitions for the themes. Kruskal-Wallis tests were conducted to assess the effect of each theme on outbreak duration, attack rate and the number of outbreaks.

### Ethics approval

Ethical exemption for this audit with intent to publish and a waiver of informed consent for access to information contained within the WBPHU data repository was granted by the Human Research Ethics Committee at Central Queensland Hospital and Health Service on 10/08/2022 (EX/2022/87,765).

## Results

### Descriptive statistics

There was a total of 55 COVID-19 outbreaks in 27 RACFs across Wide Bay during the specified 8-month period in 2022. All but one very small RACF met the definition for at least one COVID-19 outbreak, indicating a median of 2 outbreaks per facility.

Table 1 shows the outbreak outcomes, both overall and per wave. During the first wave, there was a total of 13 COVID-19 outbreaks. This was higher during the second and third waves, with 20 and 22 COVID-19 outbreaks during each period respectively. Outbreak duration (median, IQR) was highest during the second wave (23.50, 15–32) compared to the first (21, 13–25) and third waves (19.5, 13–29), with the longest outbreak at 48 days. In contrast, resident attack rate (median, IQR) was higher during the third wave at (21.34%, 5.69–31.47%) compared to the second (16.89%, 8.25–31.39%) and first wave (10.34%, 5.58–21.78%). Attack rate was mostly higher for residents than for staff, and maximum attack rate for residents was 83.33% and for staff was 47.22%. Of the 980 resident infections, there were a total of 43 COVID-19 related deaths, indicating a 4.39% case fatality rate. The index case within the facility was most often a staff member (69.10%) compared to a resident (30.90%). Around half (53.7%) of outbreaks involved memory support units and approximately half of facilities were using surgical masks as baseline PPE at the time of outbreak onset (56.36%), compared to N95 masks (43.64%).

**Table 1:** Outbreak outcomes (n = 55)

Median (IQR)	Duration (days)	Resident attack rate (%)	Staff attack rate (%)	Total attack rate (%)	CFR (%)
**Overall** (n = 55)	21 (13–32)	20 (6.38–35.71)	13.56 (6.67–24.27)	16.57 (5.70-30.54)	0 (0-8.93)
**Wave 1** (n = 13)	21 (13–25)	10.34 (5.58 21.78)	10.19 (7.32 21.70)	11.11 (4.10 17.98)	9.09 (0.00 11.11)
**Wave 2** (n = 20)	23.50 (15–32)	16.89 (8.25–31.39)	24.08 (7.82–35.44)	13.36 (7.08–23.54)	0 (0-5.57)
**Wave 3** (n = 22)	19.50 (13–29)	21.34 (5.70 31.47)	22.60 (6.00 47.76)	18.41 (5.75 31.25)	0 (0-5.5)

### Analytical statistics

Table 2 demonstrates the outbreak outcomes associated with memory support units. This had a significant relationship with both outbreak duration χ2(1) = 8.457, p = 0.0036 and attack rate χ2(1) = 24.066, p < 0.0001, including both resident attack rate χ2(1) = 24.733, p < 0.0001 and staff attack rate χ2(1) = 19.808, p < 0.0001.

**Table 2:** Outbreak outcomes per MSU involvement

	MSU involved (n = 29)	MSU not involved (n = 26)	p-value	chi^2^
**Duration (days)**	26 (20–34)	16 (12–21)	p = 0.0036	(1) = 8.457
**Resident attack rate (%)**	33.96 (21.88-50)	6.19 (2.76–10.19)	p = < 0.0001	(1) = 24.733
**Staff attack rate (%)**	21.57 (15.85–32.26)	6.54 (3.54–11.11)	p = < 0.0001	(1) = 19.808
**Total attack rate (%)**	27.39 (17.93–35.29)	5.64 (3.11-11.00)	p = < 0.0001	(1) = 24.066
**CFR**	3.92 (0-9.52)	0 (0-1.72)	NS	(1) = 3.074

Spearman’s correlation demonstrated a significant correlation between facility size and overall attack rate (=-9686, p < 0.001), including both residents (r=-0.3562 p = 00.76) and staff (r=-0.3304, p = 0.0138).

### Thematic analysis

Thematic analysis of the WBPHU outbreak documentation produced 6 themes. Quotes which best represent the essence of the subthemes are demonstrated as either barriers or enablers in Table 3.

**Table 3:** Themes and subthemes derived from thematic analysis of WBPHU outbreak documentation

Themes	Subthemes	Quotes – Enablers	Quotes - Barriers
Resources	RATs	‘Over 1000 RATs’	‘RATs reserved for symptomatic cases’
	PPE	‘Adequate PPE supply’	‘Delayed access to PPE even with known cases’
	Antivirals	‘Recent supply of antivirals largely untouched’	‘Difficulty accessing prescriptions for antivirals’ ‘Ran out of antivirals due to recent outbreak’
Communication	PHU	‘Routine daily update from facility…’	‘Difficult to contact facility, sent emails and left voicemail’
	External stakeholders	‘daily contact with COVID virtual ward’	‘Case acquired in hospital… facility unaware of [need to] isolate’
Case detection	Symptom monitoring	‘Checklist of symptoms performed routinely’	‘Difficult to distinguish COVID-19 symptoms from COPD’
	Detection of cases	‘Low threshold to test residents if symptomatic’	‘Staff working while symptomatic from recent booster vaccination’ ‘Asymptomatic staff working while infectious’ ‘Multiple residents refused testing’ ‘Resident mildly symptomatic but low CT value’ ‘Unsure who was present in dining room with [case]’ ‘New resident case… retested when actually historic case’
	RAT vs. PCR	‘Staff RATs prior to each shift’ ‘Symptomatic resident tested immediately via RAT’ ‘RATs for [close contacts] commenced immediately’	‘Symptomatic staff attending work if RAT negative’ ‘[staff] reported RAT positive but PCR negative’ ‘Resident symptomatic but RAT negative… [moved] around facility’
	Delayed results	‘PCR results received promptly [next day]’ ‘PCR testing arranged for [tomorrow]’	‘PCR results pending from [6 days prior]’ ’48 hours for Commonwealth to approve PCR testing’ ‘Swabs lost…’ ‘Unable to lift restrictions as some PCR results still pending’
Staffing	Cohorting of staff	‘Non clinical staff working from home to provide ongoing support’ ‘Staff cohorted to [area] on a permanent basis’	‘Unable to cohort due to staffing shortages’
	Workplace exclusion – close contacts and cases	‘Staff furloughed until day 8’ ‘All staff close contacts furloughed’	‘Close contacts attend work unless symptoms develop’ ‘Unsure where [staff case] worked and close contacts’
	Breaks alone	‘Staff breaks taken alone and outside’	‘Staff taking breaks with a buddy’ ‘Shared smoking breaks occurring’
	Staffing shortages	‘Staff who agree are working 12 hour shifts’	‘Staff calling in sick rather than working’ ‘Unsafe staff numbers’ ‘Agency staff not available’ ‘Multiple staff absences due to COVID or booster vaccine side effects’ ‘Majority of staff off with COVID’ ‘Extreme staff shortages… asymptomatic COVID-19 positive staff to work with positive residents’ ‘Much needed recruitment paused due to outbreak’ ‘Staff shortages at baseline… now critical’
	PPE use	‘Staff wearing N95 mask and goggles at baseline’ ‘PPE increased on [day initial case detected]’ ‘Separate donning and doffing stations’ ‘Recent PPE training for staff’	‘Staff wearing surgical masks during outbreak’ ‘PPE removed at nurses station’ ‘Staff redonning dirty masks’ ‘Removing masks when required’ (RASS) ‘Multiple PPE breaches identified in case interviews’ ‘…reinforced importance of PPE with staff’
Facility perception		‘Red zone commenced’ ‘All positive residents referred straight away to GP’ ‘Screening of close contacts has already commenced’	‘Facility asking daily if they can come out of lockdown’ [in context of ongoing transmission] ‘Staff member refused to wear PPE during night shift… looked after positive and negative residents’
Infection control	Cohorting of residents	‘Cohorting residents into positive, negative, recovered to reduce PPE burden’ ‘Cohorting residents in [name of wing/area] from [name of other wing/area]’ ‘Ceased group activities, residents having meals in rooms’	‘Due to behaviours in our dementia unit, residents unable to be cohorted’
	Isolation	‘[case] prefers to stay in their room’ ‘has not been outside his room in days’	‘Resident wandered into the room of a positive case… getting into PPE bins’ ‘[case] has been sitting in corridor’ ‘Doors open [of positive case’s room]’ ‘Visitors to positive cases’
	Cleaning	‘Regular disinfection… signage for high touch areas’ ‘Extra bins supplied and daily emptying of bins’	

#### Resources

Availability of RATs, PPE and antivirals was documented for most outbreaks. Ample supplies of RATs were observed to lead to a lower threshold for surveillance testing. Adequate resourcing and discussion around antiviral supplies were more common in subsequent facility outbreaks.

#### Communication

Frequency of communication with the PHU ranged from multiple times per day to twice per week. Most facilities emailed the PHU a prompt line list when new cases were detected. Facilities who were difficult to contact or unable to provide case details delayed PHU advice pertaining to testing and restrictions. Engagement with external stakeholders also varied, particularly communication efficacy with Commonwealth case managers and referrals to treating medical practitioners.

#### Case detection

Common symptom monitoring strategies were twice daily COVID-19 observations to monitor for temperature spikes or a daily symptom checklist provided to residents. Barriers to symptom detection included difficulty differentiating COVID-19 symptoms from chronic respiratory conditions and asymptomatic residents and staff, commonly observed after recent COVID-19 booster vaccine. Inadequate staffing prevented thorough symptom monitoring during some outbreaks, leading to reliance on observation of more overt symptoms such as coughing.

Case detection was supported with widespread RAT availability within facilities. Many facilities had a low threshold for testing in the presence of any symptoms. Most facilities had a staff surveillance testing protocol and some offered routine RATs for residents. Barriers to case finding included resident refusing testing, insufficient details available for contact tracing and confusion surrounding COVID-19 reinfections due to multiple guideline changes regarding testing of historic cases.

Testing was performed via both RAT and PCR during most outbreaks. RATs were largely observed to be an effective tool for prompt case finding, although issues were encountered with some misunderstanding results as definitive, with periodic false negative results. PCR testing was observed as useful for identifying asymptomatic cases and reducing the burden on facility staff performing RATs on residents. However, during peak COVID-19 waves, PCR testing was associated with delays in arranging a test date and receiving results.

#### Staffing

Cohorting of staff refers to separating areas of the facility into exposed and unexposed, then limiting staff to only work in one section. Some facilities engage in this set up on a permanent basis, while others were unable to implement this at all.

Workplace exclusion for staff COVID-19 cases occurred in majority of outbreaks, with confirmed cases only required to attend work in extreme staff shortages and appropriate precautions in consultation with the PHU. Some facilities were able to furlough close contacts as well as cases. Shared breaks between staff were identified as a source of transmission during some outbreaks, while other facilities enforced staff to have breaks alone.

Staff shortages were a concern during most outbreaks leading to frequent overtime, reliance on agency staff and in some cases, escalation to a state surge workforce or even the Australian Defence Force. Low staffing levels posited a barrier to many other themes including communication, case detection and staff cohorting.

PPE use was identified as a common strategy which either enabled outbreak control or potentiated transmission. PPE breeches, largely pertaining to mask use, were often established as the likely source of transmission between staff and residents. PHU case interviews highlighted staff breaches such as removing masks when clinically indicated, failing to change PPE throughout shifts and redonning used PPE. Masks were commonly removed to enable communication with a resident, or whilst socialising with other staff in office areas. Baseline PPE was documented for all outbreaks. In contrast, appropriate PPE use was established as an effective approach to mitigate transmission risk, including PPE education refresher training, prompt upgrade to N95 masks upon outbreak detection, combined with strict donning and doffing protocols.

#### Facility perceptions

Perceptions of COVID-19 and the need for a thorough outbreak response differed between outbreaks and facilities. Most facilities shared the PHU understanding of the risks to residents posed by COVID-19, as indicated by effective collaboration and coordination of a sustained outbreak response. During subsequent outbreaks, facilities were largely proactive in testing and isolating prior to even notifying PHU. In contrast, there was a poor understanding of the rationale for immediate risk assessment and associated restrictions during some outbreaks. This was observed at both a management level, as well as staff caring for residents, which raised concerns for the efficacy of implementing PHU advice.

#### Infection control

Isolation of COVID-19 residents was prioritised across all outbreaks. Most facilities encouraged close contact residents to remain within their exposed section of the facility, to minimise movement into unexposed areas. Large group activities and communal meals were often paused due to potential transmission risk. The main barrier to adequate isolation of residents was wandering behaviours, often reported in outbreaks involving memory support units, as well as some facilities who permitted visitors to attend COVID-19 positive residents during their isolating period. Regular surface disinfection, hand hygiene and waste management were common cleaning strategies. Appropriate signage around the facility was reported to the PHU as aiding staff, resident and visitor understanding and compliance.

### Framework analysis

Further review of the outbreak documentation was completed via a framework analysis, as per the rating system detailed in Fig. 1.

Tables 4 and 5 show the impact of each theme on overall attack rate and outbreak duration respectively. Outbreak duration was statistically significant between the extent of staff shortages: no staffing issues (n = 8), low staffing during outbreak (n = 30) and extreme staffing issues requiring escalation (n = 11), adjusted for ties, χ2(3) = 9.710, p = 0.0212.

**Table 4:** Overall attack rate, per theme

**Theme**	**Median (IQR)**					Attack rate	
						p-value	χ2
	Limited	Good	Ample				
**RAT availability**	19.37 (10.035–38.73)	27.11 (3.56–38.61)	15.22 (5.84-30)			NS	(2) = 0.285
**PPE availability**	16.96 (3.11–21.78)	27.39 (9.49–38.61)	13.86 (4.74–21.89)			NS	(2) = 3.976
	Delayed	Next day	Same day				
**Communication**	21.78 (7.01–27.39)	27.55 (10.34–36.76)	8.58 (4.06–17.93)			0.0175	(2) = 8.093
**Detection of cases**	23.96 (10.04–27.25)	24.42 (9.92–39.75)	13.77 (4.06–20.79)			0.0296	(2) = 7.042
	None	Mostly	Complete				
**Staff cohorting**	31.47 (21.52–50.59)	14.21 (5.17–27.11)	11.22 (3.77–18.87)			0.0073	(2) = 9.831
	Cases mostly excluded	Cases fully excluded	Close contacts excluded				
**Staff exclusion**	27.11 (16.96–56.90)	16.20 (5.70-30.54)	3.85 (1.03–6.67)			NS	(2) = 4.741
	Extreme shortage	Moderate shortage	No shortage				
**Staff shortages**	38.61 (21.78–51.67)	17.72 (8.23–27.39)	3.39 (2.56–6.13)			< 0.0001	(2) = 19.98

NS = not significant.

**Table 5:** Outbreak duration, per theme

**Theme**	**Median (IQR)**				Duration	
					p-value	χ2
	Limited	Good	Ample			
**RAT availability**	17 (12.5–23)	23 (12–34)	20 (14–32)		NS	(2) = 1.013
**PPE availability**	13 (12–25)	23 (19–25)	20 (14–32)		NS	(2) = 0.845
	Delayed	Next day	Same day			
**Communication**	24 (12–32)	18 (13–23)	23 (16–39)		NS	(2) = 2.544
**Detection of cases**	22.5 (12.5–32.5)	20.5 (13.5–26)	20 (14–34)		NS	(2) = 0.375
	None	Mostly	Complete			
**Staff cohorting**	21 (14–34)	20 (13–29)	21 (16–34)		NS	(2) = 0.490
	Cases mostly excluded	Cases fully excluded	Close contacts excluded			
**Staff exclusion**	14 (12–24)	21 (14–32)	13.5 (11–16)		NS	(2) = 3.202
	Extreme shortage	Moderate shortage	No shortage			
**Staff shortages**	25 (18–34)	23 (16–34)	14.5 (11-19.5)		0.0370	(2) = 6.573

NS = not significant.

There was a significant relationship between the overall attack rate and communication (p = 0.016), detection of cases (p = 0.027), staff cohorting (p = 0.018) and staff shortages (p < 0.001).

## Discussion

To the best of our knowledge, this is the first review of RACF COVID-19 outbreaks in Australia. We took advantage of all available outbreak data collected by WBPHU during 2022, which provided a clear overview of the RACF outbreaks associated with the three COVID-19 waves in Queensland to date.

The number and severity of RACF outbreaks generally reflected the extent of COVID-19 transmission within the community. [24] The overall attack rate of 26% for residents concurred with previous findings which established a pooled attack rate of 28%. [25, 26] Furthermore, the lower case fatality rates in aged care during subsequent COVID-19 waves is also consistent with international literature, where the contribution of COVID-19 deaths from aged care to total mortality reduced in subsequent waves of the pandemic. [27, 28] Previous studies have identified an association between facility size and COVID-19 attack rate, which was also replicated in our comparatively smaller sample. [29]

This review provides practical learning points for PHUs to support RACFs in future outbreaks. Most notably, it promotes the need for communication efficiency and regularity between RACFs and external stakeholders. While some RACFs notify the PHU of outbreaks early and provided daily updates via email and phone, delayed contact with facilities renders timely public health intervention a significant challenge. [30, 31] Relationships between RACFs and PHUs have likely been strained throughout 2022, particularly when public health advice differed from facility outbreak guidelines. A goal for PHUs work towards rebuilding relationships with RACFs for ongoing engagement in not only COVID-19 outbreak management, but all communicable disease control and health promotion. [31]

Prompt case detection is imperative to prevent further spread, especially when exposed staff or residents may act as a conduit to further cases. prompt symptom monitoring is crucial and RACFs should remain vigilant following exposure as symptoms may develop up to 14 days following exposure. [32] While some literature has suggested the role of surveillance testing outside of outbreaks [33, 34], this has limited efficacy to justify the time burden and staff testing protocols vary between facility. It is important RACFs understand what constitutes symptom monitoring, while also recognising the possibility of asymptomatic cases. [9, 19]

Aged care across Australia has a workforce deficit at baseline [35, 36], so this is further exacerbated when large numbers of staff are furloughed due to COVID-19. [37] Reliance on agency staff is suboptimal for both staff transmission [38] and resident outcomes [39] but even this resource was limited during the peak of each COVID-19 wave, with multiple facilities facing concurrent staffing shortages. [40] While this is a systemic issue beyond the scope of PHUs [8, 41], it is crucial to remain mindful of both acute and chronic staffing concerns, given the implications it has on the feasibility of desired risk mitigation strategies.

Shared breaks among staff were another common occurrence among facilities, which posed the risk of further propagating transmission. During an active COVID-19 outbreak, staff should be encouraged to take breaks alone, to ensure their PPE remains in situ during all interactions within the facility. [42] The risk of staff infections has been shown as greater during an outbreak when compared to a COVID-19 ward, which may be in part associated with hand hygiene and PPE compliance [43]. The attack rate is higher for staff who work closely with residents, such as personal care workers or nursing assistants, when compared to other healthcare workers. [11, 44] While PHUs often advise on the specific PPE required, there is clearly a need to encourage education on donning and doffing protocols, as well as reinforcing the importance of regular and adequate hand hygiene. [16, 45]

With cessation of the requirement for close contacts to isolate on the 28th of April 2022 [46], management of COVID-19 exposed residents and staff became a key responsibility of RACFs. Few RACFs had the resources to furlough close contact staff during their incubation period, with exposed staff largely required to attend work which thus posed a risk of internal transmission. RACFs who allocated staff to work within a single area were often able to prevent spread to multiple areas. [16, 45] Interestingly, some facilities have adopted staff cohorting as a permanent practice to effectively contain any COVID-19 transmission within limited sections of the facility.

Equally, cohorting exposed residents was another common strategy, although raises ethical concerns regarding the imposition of movement restrictions on liberty. Public health, especially communicable disease control, balances risk and benefit, so outbreak management must consider quality of life, freedom and in the context of circulating infections. [12, 13] Failure to recognise the utmost importance of resident social interaction can have a detrimental impact on elderly mental health, particularly if visitors are limited during outbreaks. [47] Consultation to identify those most at risk of social isolation and physical decline [48], COVID-19 safe methods to facilitate physical activity and implementing ‘lifestyle’ staff to engage with residents using appropriate precautions has been a novel and efficacious strategy to support wellbeing. [49]

Poor resident compliance with isolation and wandering behaviours propagates further transmission and a likely contributor to the frequency of outbreaks among memory support units. [38] Indeed, dementia is a risk factor for contracting COVID-19 due to impaired cognition and memory preventing adequate understanding of the outbreak. [14] This can result in poor compliance with recommended public health intervention, such as poor hand hygiene, social distancing, wandering and delayed recognition of symptoms. [50–53]

### Strengths

The long timeframe covered in this study allows for a well-rounded analysis of barriers and enablers to outbreak management across multiple different time points and how these evolved with the different COVID-19 waves in Queensland.

Despite the context of this review situated in regional Queensland, there are no identified nuances of these outbreaks, and the findings are likely generalisable to other RACFs across Australia.

### Limitations

As a retrospective review, the main limitation encountered was incomplete documentation. PHU documentation varied between clinical staff which resulted in some missing values across some themes. This review also only captures those outbreaks reported to the PHU and therefore any outbreaks that went unreported will be missed creating a reporting bias. While we initially planned to collect data on resident hospitalisation rates, complete records of this information for all RACFs were not readily available to the WBPHU. Initially all positive cases were referred to the COVID-19 virtual ward, however with the availability of antivirals on the Pharmaceutical Benefits Scheme (PBS), the majority of RACF residents now receive medical follow up through their regular general practitioner.

Vaccination status was only available during the third wave. While this review did not focus on vaccination data, it would be remiss to acknowledge its importance as an enabler of effective outbreak management. [26, 54] This is especially important for the elderly, who have a weaker vaccine response lending to breakthrough infections, as well as newer COVID-19 variants deemed to be capable of evading vaccine immunity. [54]

Moreover, the findings in this review are derived solely from a PHU perspective, without any external stakeholder consultation. Further research would be beneficial, with input from RACFs to assess if their perspectives corroborate these findings as important aspects of COVID-19 outbreak management.

## Conclusion

Effective outbreak management strategies are imperative to address COVID-19 in aged care and contingent on resources required for testing, infection control and treatment. By acknowledging the health inequities that elderly people face with higher COVID-19 case fatality rates, regular reflection of outbreaks can improve RACF preparedness and response to inform best practice guidelines for protection of aged care residents and staff. [20] While most RACFs have now experienced at least one COVID-19 outbreak, PHUs still play an important role in guiding facilities to implement appropriate testing and restrictions. Regular communication enables timely advice and clarification when required, while ongoing education from PHUs can support RACFs to undertake prompt case finding and staffing strategies to mitigate the extent of facility transmission. COVID-19 is not over, nor should a collaborative approach and continuous improvement towards thorough outbreak management.

## Data Availability

The datasets used and/or analysed during the current study are available from the corresponding author on reasonable request.

## List of abbreviations

PHU: Public Health Unit
RACF: Residential Aged Care Facility
CDNA: Communicable Disease Network Australia
RAT: Rapid Antigen Test
PCR: Polymerase Chain Reaction
PPE: Personal Protective Equipment
MSU: Memory Support Unit
PBS: Pharmaceutical Benefits Scheme
